# Connexins in epidermal health and diseases: insights into their mutations, implications, and therapeutic solutions

**DOI:** 10.3389/fphys.2024.1346971

**Published:** 2024-05-07

**Authors:** S. Suheda Yasarbas, Ece Inal, M. Azra Yildirim, Sandrine Dubrac, Jérôme Lamartine, Gulistan Mese

**Affiliations:** ^1^ Izmir Institute of Technology, Faculty of Science, Department of Molecular Biology and Genetics, Izmir, Turkiye; ^2^ Department of Dermatology, Venereology and Allergology, Medical University of Innsbruck, Innsbruck, Austria; ^3^ Skin Functional Integrity Group, Laboratory for Tissue Biology and Therapeutics Engineering (LBTI) CNRS UMR5305, University of Lyon, Lyon, France

**Keywords:** connexins, epidermal homeostasis, skin disorders, mutations, dysregulation, therapeutic approaches

## Abstract

The epidermis, the outermost layer of the skin, serves as a protective barrier against external factors. Epidermal differentiation, a tightly regulated process essential for epidermal homeostasis, epidermal barrier formation and skin integrity maintenance, is orchestrated by several players, including signaling molecules, calcium gradient and junctional complexes such as gap junctions (GJs). GJ proteins, known as connexins facilitate cell-to-cell communication between adjacent keratinocytes. Connexins can function as either hemichannels or GJs, depending on their interaction with other connexons from neighboring keratinocytes. These channels enable the transport of metabolites, cAMP, microRNAs, and ions, including Ca^2+^, across cell membranes. At least ten distinct connexins are expressed within the epidermis and mutations in at least five of them has been linked to various skin disorders. Connexin mutations may cause aberrant channel activity by altering their synthesis, their gating properties, their intracellular trafficking, and the assembly of hemichannels and GJ channels. In addition to mutations, connexin expression is dysregulated in other skin conditions including psoriasis, chronic wound and skin cancers, indicating the crucial role of connexins in skin homeostasis. Current treatment options for conditions with mutant or altered connexins are limited and primarily focus on symptom management. Several therapeutics, including non-peptide chemicals, antibodies, mimetic peptides and allele-specific small interfering RNAs are promising in treating connexin-related skin disorders. Since connexins play crucial roles in maintaining epidermal homeostasis as shown with linkage to a range of skin disorders and cancer, further investigations are warranted to decipher the molecular and cellular alterations within cells due to mutations or altered expression, leading to abnormal proliferation and differentiation. This would also help characterize the roles of each isoform in skin homeostasis, in addition to the development of innovative therapeutic interventions. This review highlights the critical functions of connexins in the epidermis and the association between connexins and skin disorders, and discusses potential therapeutic options.

## Introduction

The skin serves as a barrier, protecting the human body from external threats. Primary functions of skin tissue include preventing water loss, regulating temperature, forming a barrier to facilitate molecular exchange and maintaining immune surveillance (García-Vega et al., 2019; Anderton and Alqudah, 2022). There are three layers in the skin; the epidermis, the dermis and the hypodermis with the epidermis and dermis separated by the dermoepidermal junction, including a basal lamina (Czyz et al., 2023). The epidermis, the outermost layer of the skin, is an avascular tissue and is composed primarily of keratinocytes (Faniku et al., 2015; García-Vega et al., 2019). This layer is further subdivided into four distinct layers, each with specific features, starting from the innermost layer to the outermost, i.e., the stratum basale, the stratum spinosum, the stratum granulosum, and the stratum corneum. The basal layer contains proliferating keratinocytes that are able to divide to produce committed cells migrating towards the upper layers of the epidermis and undergoing a differentiation process leading to a specialized terminally state named corneocyte (Fuchs, 2008; Martin et al., 2014; Faniku et al., 2015). During epidermal differentiation, keratinocytes undergo significant modifications in both protein expression and structural organization, which are orchestrated by complex signaling pathways, basement membrane components, calcium balance, and adhesion molecules (Fuchs, 2008). In addition, a calcium ion (Ca^2+^) gradient exists within the epidermis, increasing from the basal layer to the granular layer (Tu et al., 2012; Adams et al., 2015). This Ca^2+^ gradient plays a crucial role in a plethora of cellular pathways during keratinocyte differentiation process. Moreover, both intracellular and extracellular Ca^2+^ regulates signaling pathways as well as the expression and function of adhesion molecules such as integrins that connect keratinocytes to each other and to the basement membrane (Pani and Singh, 2008). The Ca^2+^ gradient is critical for keratinocyte differentiation and skin barrier formation through activity of transcription factors such as activator protein 1 (AP-1) that induces keratinocyte-specific differentiation genes such as involucrin and profilaggrin (Ng et al., 2000). Moreover, Ca^2+^ signaling has been shown to preserve the integrity of both the physical and anti-bacterial barrier of the stratum corneum by regulating lamellar body secretion and cytokine expression, respectively (Choi and Yoon, 2014; Lee and Lee, 2018; Srinivas et al., 2018).

Because the epidermis lacks blood vessels, the coordination and the communication among epidermal cells are ensured by intercellular junctions. One crucial mechanism for this communication is gap junctions (GJs), which are specialized connections enabling the direct intercellular communication between cytoplasm of adjacent cells. In this article, we will explore into the formation and regulation of the GJ channels, specifically examining the functions and the consequences of gene mutations in connexin 26 (Cx26), Cx30, Cx30.3, Cx31, and Cx43, which have been linked to various skin disorders. Moreover, we will provide a concise overview of the dysregulation of connexins in other dermatological conditions, offering valuable insights into the role of connexins in maintaining healthy skin and the essential role of gap junctional intercellular communication (GJIC) in this process.

## Connexins and gap junctions

**FIGURE 1:**
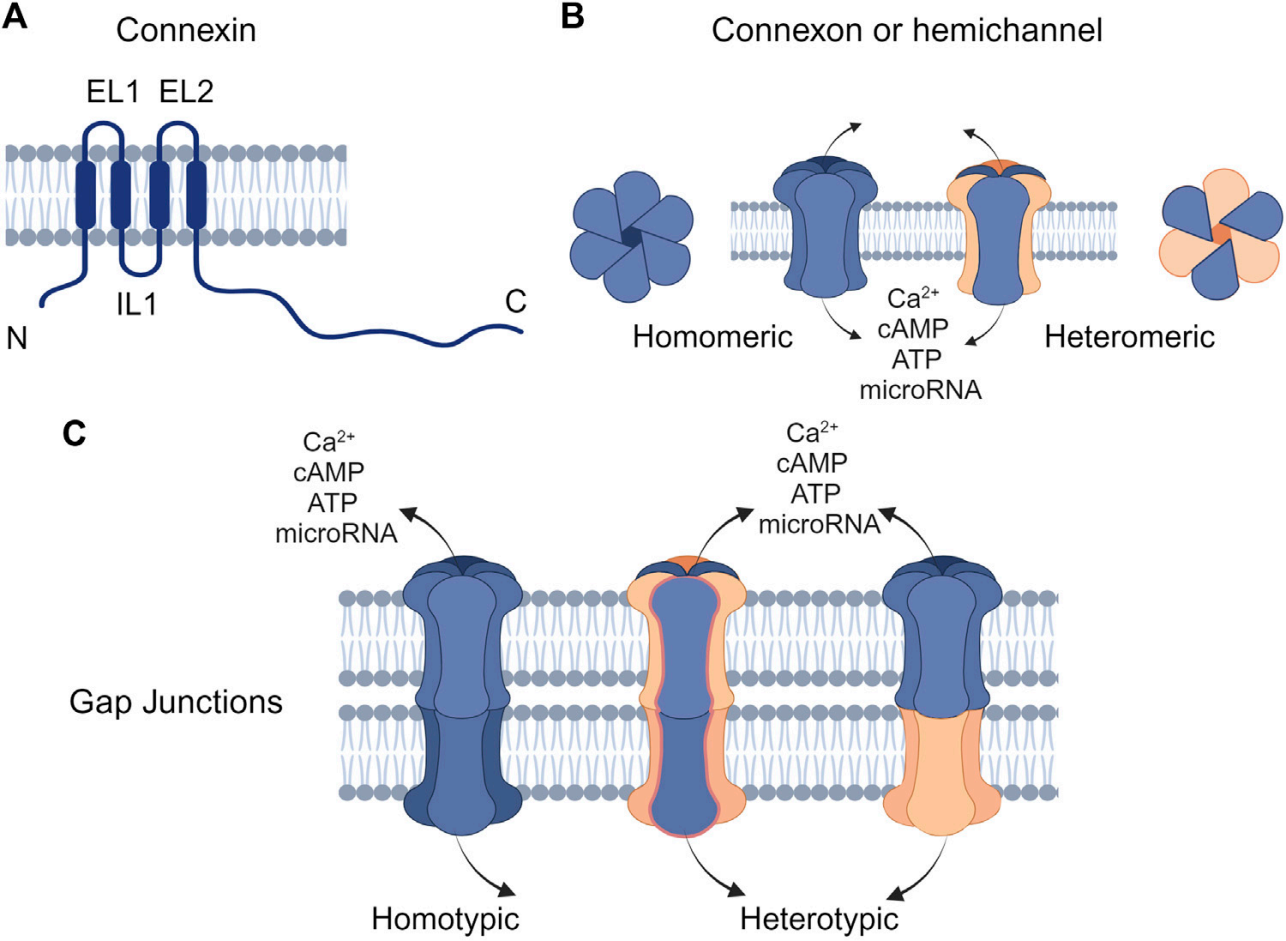
Structure of connexins and gap junctions (GJs) **(A)** Connexins are composed of four transmembrane domains (TM1-TM4) with intracellular N- and C-terminal domains and two extracellular loops (EL1 and EL2). **(B)** Connexins can oligomerize to form hemichannels, which can be either homomeric (composed of a single type of connexin) or heteromeric (composed of different types of connexins). **(C)** Hemichannels on the plasma membrane can interlock with connexons of the same or different types on the cell membrane to form homotypic or heterotypic GJ channels, respectively. Created with BioRender (2023).

GJs facilitate the transport of ions (Ca^2+^ and K^+^), small metabolites (glucose, vitamins, and amino acids), secondary messengers (cAMP and ATP), and short interfering RNAs between neighboring cells (Figure 1) (Goodenough and Paul, 2009; Aypek et al., 2016; Garcia-Vega et al., 2021). GJs are formed by connexins (Figure 1A) that assemble into hemichannels or connexons (Figure 1B), which then can dock with connexons from neighboring cells to mediate GJIC on the plasma membrane (Figure 1C). This intercellular communication plays a pivotal role in numerous physiological processes, including cell proliferation, differentiation, and homeostasis (Anderton and Alqudah, 2022). Consequently, any disruption in connexin function or GJIC can lead to diseases.

**FIGURE 2:**
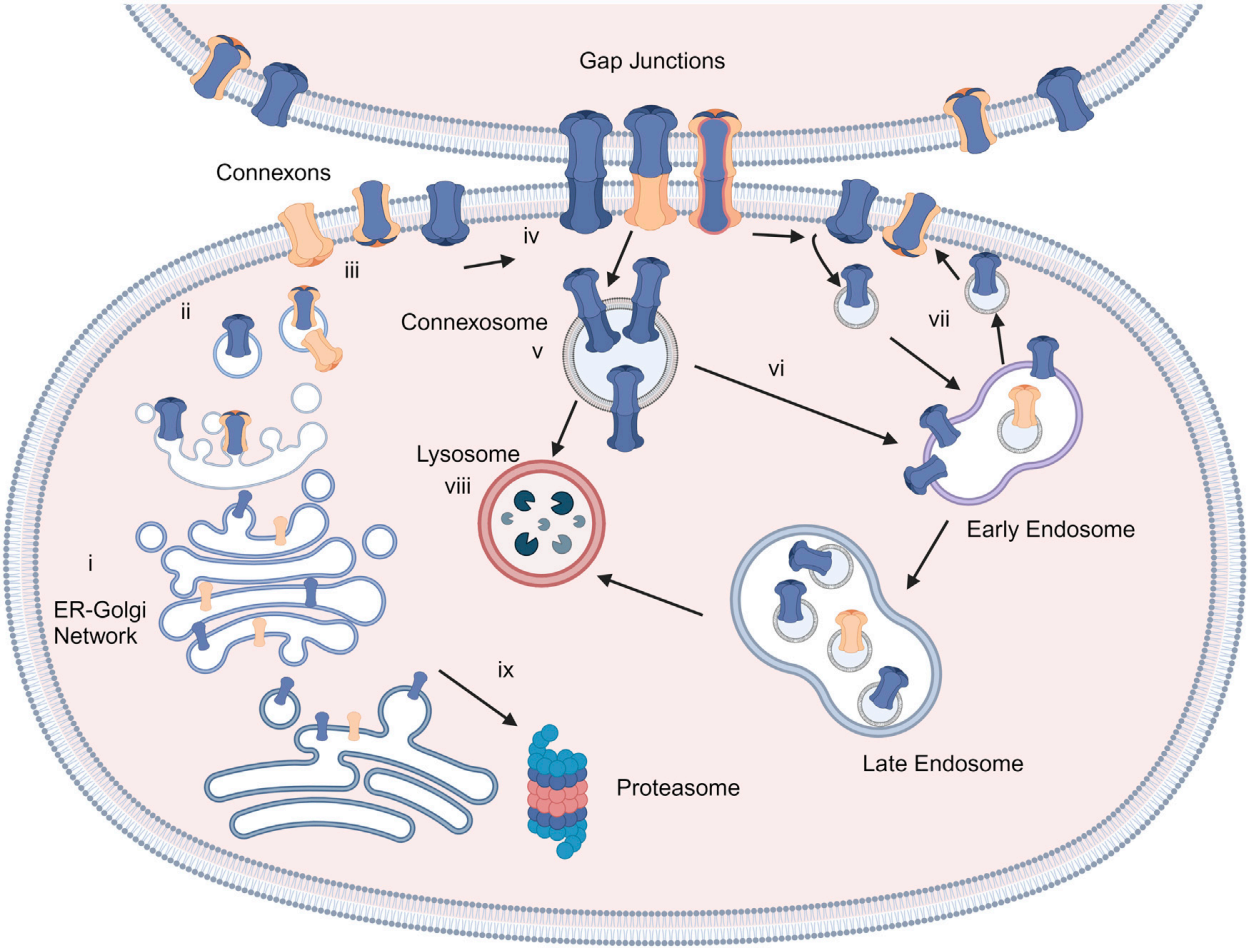
Connexins and GJ biosynthesis. Connexins are inserted into the endoplasmic reticulum-Golgi network **(i)** and can be degraded in proteasomes **(ix)**. They assemble into connexons/hemichannels and transported to the plasma membrane **(ii, iii)**, where they can form GJ channels with adjacent cells **(iv)** or function as individual hemichannels on the membrane **(iii)**. GJs can be endocytosed to create connexosomes **(v)**, which may be degraded by endosome-lysosome route **(vi)**. Connexins are sorted from early endosomes to lysosomes **(viii)** or recycled to the plasma membrane **(vii)**. Created with BioRender (2023).

Connexins are the main structural subunits of GJ channels in chordates, and possess an intracellular loop (IL), an amino (N) and a carboxyl (C) domains, along with four conserved transmembrane helical domains and two extracellular loops (EL1 and EL2) (Figure 1A) (Martin and van Steensel, 2015). Connexons are formed in the endoplasmic reticulum (ER)—Golgi network through the oligomerization of six connexins, which can assemble either into homomeric hemichannels, composed of the same type of connexins, or heteromeric connexons, assembled by different types of connexins (Figure 2i). Following trafficking to the plasma membrane, hemichannels can dock head-to-head with other hemichannels from neighboring cells to complete the formation of the GJs, hence facilitating the intercellular communication (Figure 2ii-iv). Hemichannels can also function independently in non-junctional areas, mediating the exchange of molecules across the plasma membrane (Figure 2iii). Additionally, connexins can influence gene expression, cellular migration, and other molecular mechanisms by localizing to the cytoplasm and the nucleus without forming channels on the plasma membrane (Kotini et al., 2018; Martins-Marques et al., 2023). Connexins have a short half-life and the turnover of the hemichannels and GJs, which is mediated by endocytosis at the plasma membrane, enables cells to rapidly respond to environmental changes (Figure 2) (VanSlyke and Musil, 2005; Falk et al., 2016; Aasen et al., 2019). After GJ endocytosis, connexins follow various intracellular routes before lysosomal degradation (Figures 2v–2viii). These routes include fusion of connexosomes with early endosomes (Figure 2vi), enclosure by autophagosomes followed by fusion with lysosomes, and direct fusion with lysosomes (Figure 2viii) (Carette et al., 2015; Totland et al., 2020).

GJs regulate cellular homeostasis by facilitating the transport of molecules with molecular weights below 1.4 kDa (Sanchez and Verselis, 2014; Leybaert et al., 2017; Bai et al., 2018). Due to their critical functions, connexin expression and channel activity are tightly regulated by various factors, including extracellular Ca^2+^, intracellular pH, membrane potential, post-translational modifications, and epigenetic regulation (Aasen et al., 2018; 2019). Moreover, non-coding RNAs including microRNAs can also regulate connexin expression (Curcio et al., 2013; Zeng et al., 2021). Extracellular Ca^2+^ exerts a significant influence on connexin channel gating by directly binding to the channels. For example, high Ca^2+^ concentrations constrict the diameter of the Cx26 channel pore lumen (Lopez et al., 2016), maintaining these hemichannels in a predominantly closed state (Chen et al., 2022). Cx26 and Cx30 hemichannels possess specific residues, such as D50 and K61, which form salt bridges at the extracellular entrance of the pore. Ca^2+^ binding to these residues disrupts the salt bridge, promoting the closed state of Cx26 and Cx30 hemichannels (Lopez et al., 2016). Connexins, especially Cx26, display a distinct distribution of charged amino acid residues: the cytoplasmic domain is positively charged, while the transmembrane and extracellular domains are negatively charged (Maeda et al., 2009). Within the pore lining, negatively charged residues form an electrostatic network that is essential for hemichannel gating and Ca^2+^ binding. Beyond these direct effects, Ca^2+^ regulates its own transport by interacting with the Ca^2+^ binding protein, namely, calmodulin. Calmodulin binding was shown to induce Cx43 channel closure, indicating the involvement of Ca^2+^/calmodulin mechanisms in channel gating (Xu et al., 2012).

Connexin channels exhibit sensitivity to changes in environmental pH. A decrease in pH triggers the closure of Cx43 channels, a process mediated by conformational changes in the cytoplasmic domains, which act as gating plugs in response to acidic conditions (Peracchia, 2004). Similarly, channel closure under acidic conditions is observed in Cx26 due to conformational changes (Wang et al., 2012). In this instance, the pore lumen is occluded through voltage-driven alterations in its N-terminal domain (Maeda et al., 2009; Brotherton et al., 2022). Moreover, voltage-dependent conformational changes serve as a crucial regulator of connexin channel gating which is induced by a potential difference either between the extracellular and intracellular spaces or between the cytoplasm of neighboring cells (Bargiello et al., 2012; Oh and Bargiello, 2015). For example, Cx26 hemichannels become active at −50 mV, with their activation increasing upon depolarization until they become inactive at positive membrane potentials (González et al., 2006). Voltage-driven alterations in the shape of ion channels, achieved by modifying the positions of charged amino acids, are linked to other structural alterations including destabilization of channel pore parahelix segment that determine whether the channel pore is open or closed (Bargiello et al., 2018).

Connexins harbor multiple sites susceptible to post-translational modifications, such as phosphorylation, S-nitrosylation, and SUMOylation, which can modulate channel functions in diverse ways. Phosphorylation of serine, threonine, or tyrosine residues within connexins can influence their assembly and trafficking, whereas S-nitrosylation induces structural changes in connexin proteins by altering the cysteine oxidation state (Saez et al., 2003). Small ubiquitin-like modifier (SUMO) proteins can conjugate with lysine residues of connexins and regulate GJ conformation and trafficking (Kjenseth et al., 2012; Aasen et al., 2018). Furthermore, connexin expression can be regulated through epigenetic mechanisms and dysregulation of epigenetic processes is indicated in various diseases (Vinken, 2016; Aasen et al., 2018). For example, Cx43 downregulation in breast cancer was attributed to the hypermethylation of its promoter region (Yi et al., 2007). In addition, inhibition of histone deacetylase caused the elevation of Cx43 expression and facilitated its translocation to the plasma membrane in tumorigenic cells (Oyamada et al., 2005), highlighting the involvement of epigenetic regulatory mechanisms for control of connexin expression (Aasen et al., 2018). As briefly summarized, connexin channel function represents a complex process tightly regulated by a multitude of factors. These regulatory mechanisms ensure appropriate connexin function in response to diverse cellular and environmental cues.

## Connexins in the epidermis

**FIGURE 3:**
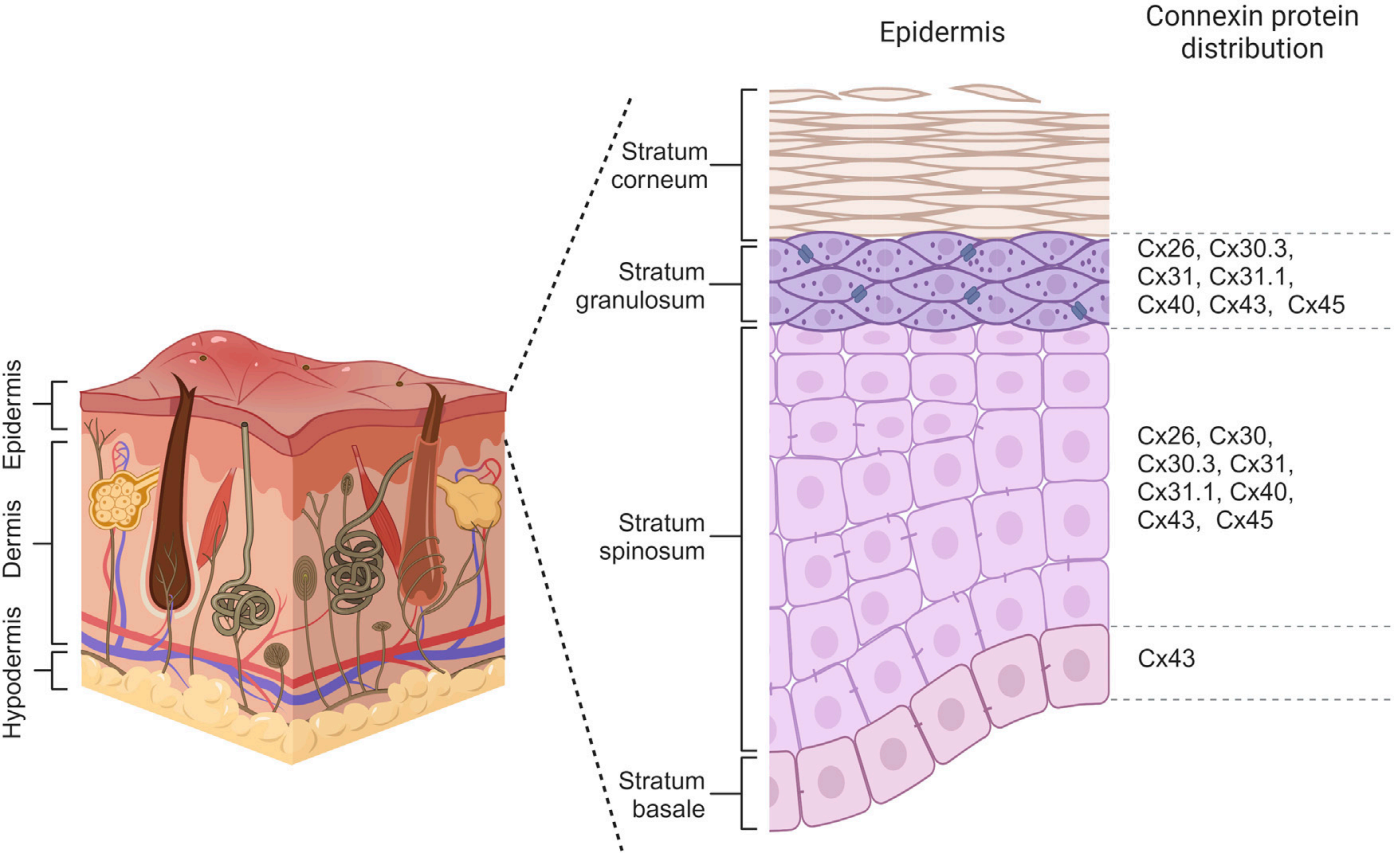
Connexin distribution within different layers of the epidermis. Basal cells primarily express Cx43 while Cx26, Cx30, Cx30.3, Cx31, Cx31.1, Cx40, Cx43 and Cx45 are observed in cells of spinosum layer. Keratinocytes of granulosum have differential expression of Cx26, Cx30.3, Cx31, Cx31.1, Cx40, Cx43, and Cx45. Created with BioRender (2023).

Connexin expression patterns in the epidermis vary considerably across its distinct layers, playing a crucial role in maintaining the epidermal homeostasis and renewing the skin. The basal layer, harboring the epidermal basal cell pool responsible for replenishing the upper cell layers, maintains a delicate balance between the rate of cell differentiation in this layer and the rate of terminally differentiated cells reaching the epidermis’ outer surface (Fuchs, 2008). Throughout the epidermal differentiation process, immunohistochemical analysis revealed that at least ten distinct connexins are expressed within the various layers of the epidermis. In the basal layer, cells primarily express Cx43, while Cx26, Cx30, Cx30.3, Cx31, Cx31.1, Cx40, Cx43, and Cx45 are observed in the spinous layer. Transitioning to the granular layer, Cx30.3, Cx31, and Cx43 are expressed at high levels, whereas Cx26, Cx31.1, Cx40, and Cx45 are found at lower levels (Figure 3) (Martin et al., 2014; Zhang and Cui, 2017). Beyond their specific distribution in the epidermis, there is a link between various skin disorders and mutations in Cx26, Cx30, Cx30.3, Cx31, and Cx43, suggesting their substantial roles in establishing and maintaining the integrity of the epidermal barrier (García-Vega et al., 2019; Lucaciu et al., 2023).

The functional significance of different connexin isoforms expressed in the epidermis is still being investigated. For example, Cx43, which is predominantly expressed in most layers, particularly the basal layer, regulates both proliferation and differentiation of keratinocytes. Cx43 interacts with junctional proteins, including the tight junction molecules, zona occludens, and adherens junction components beta-catenin, to regulate proliferation and differentiation (Scott et al., 2012). In addition, Cx26 and Cx30 channels play a regulatory role in processes such as wound healing within the granular and spinosum layers of the epidermis by influencing protein pathways associated with proliferation through the passage of molecules such as ATP and Ca^2+^ (Essenfelder et al., 2004; Srinivas et al., 2018). The precise function of each connexin isoform within the epidermis remains elusive. Therefore, unveiling the effects of skin-associated connexin gene mutations can provide valuable insights into their function in maintaining epidermal homeostasis.

## Connexin mutations in skin diseases

**FIGURE 4:**
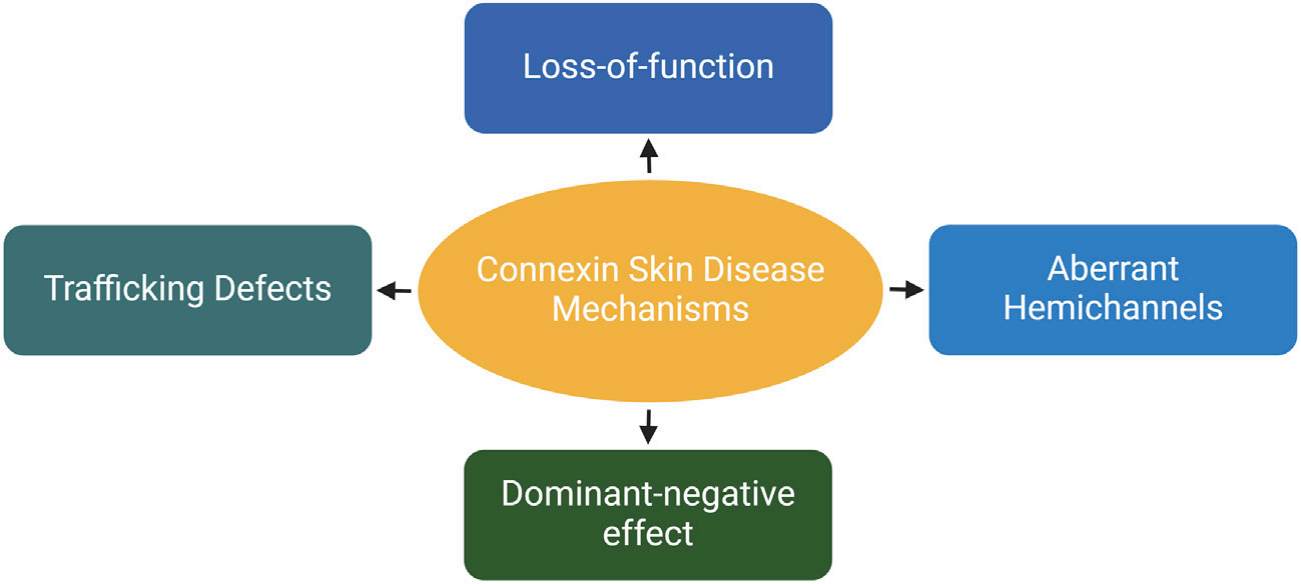
The effects of skin-associated connexin mutations on connexins and/or channels. Depending on the mutation type and location on the proteins, they can cause trafficking defects leading to retention of proteins in the cytosol. They inhibit the channel activity on the plasma membrane (loss-of-function). Additionally, mutations can have an inhibitory effects on other connexins expressed within the same cell. Finally, they can induce the dormation of hyperactive hemichannels, resulting in uncontrolled molecular exchange across the plasma membrane. Created with BioRender (2023).

The epidermis lacks blood vessels, so it relies on GJIC between keratinocytes for the coordination of signals and molecule supply to maintain epidermal homeostasis (Chanson et al., 2018; Qiu et al., 2022). Humans express at least ten distinct connexin isoforms (Mese et al., 2011; Garcia-Vega et al., 2021), and at least five of these have been implicated in eleven human skin diseases (Table 1). Six of these diseases are caused by mutations in the *GJB2* gene, which encodes for the Cx26 protein (Zhang and Cui, 2017; Sellitto et al., 2021). In addition to Cx26, mutations in *GJB6*, *GJB4*, *GJB3* and *GJA1*, encoding Cx30, Cx30.3, Cx31 and Cx43, respectively have also been linked to diverse epidermal diseases (Avshalumova et al., 2014; Laird and Lampe, 2022). Disease-associated mutations in connexin genes can disrupt GJIC in various ways, leading to diverse phenotypes. These mutations can lead to the formation of aberrant hemichannels, allowing uncontrolled passage of molecules across the plasma membrane. Mutations can also result in loss of function, where the connexin protein is unable to form functional GJs. Additionally, mutations can cause mislocalization of connexin proteins within the cell, hence preventing them from reaching the cell membrane and forming GJs. Finally, they can have a dominant-negative effect on the GJIC facilitated by other connexins (Figure 4) (Scott et al., 2012). Here, we will first discuss the Cx26-associated epidermal diseases followed by Cx30.3, Cx31, Cx43 and Cx30 skin diseases.

**TABLE 1:** Connexins, mutations and the mechanisms of mutations.

Genes	Diseases	Mutations	Inheritance	Mechanisms of Mutations	Animal Models
GJB2 (Cx26)	Palmoplantar keratoderma (PPK) with deafness (OMIM 148350)	M34K, DelE42, D66H, G59A, G59R, H73R, H73Q, R75W, G130V, S183F	Autosomal dominant	Impaired GJIC	
				Dominant-negative effect on wild-type connexins	
	Vohwinkel's syndrome (OMIM 124500)	G59S, Y65H, D66H, G130V	Autosomal dominant	Intracellular retention	Cx26-D66H mouse model
				Impaired GJIC	
	Bart-Pumphrey syndrome (BPS) (OMIM 149200)	N54K, G59S	Autosomal dominant	Intracellular retention	
				Dominant-negative effect on wild-type connexins	
	Keratitis-ichthyosis-deafness (KID) syndrome (OMIM 148210)	G11E, G12R, N14K, S17F, A40V, G45E, D50N, A88V	Autosomal dominant	Hyperactive hemichannels	Cx26-S17F mouse model
	Hystrix-like-ichthyosis-deafness (HID) syndrome (OMIM 602540)	D50N	Autosomal dominant		Cx26-G45E mouse model
GJB3 (Cx31)	Erythematokeratodermia variabilis (OMIM 133200)	L34P, V30I, G12R, G12D, R42P, G45E, C86S, F137L	Autosomal recessive	Intracellular retention	Cx31-F137L mouse model
			Autosomal dominant	ER stress	Cx31-WT overexpression mouse model
				Hyperactive hemichannels	
GJB4 (Cx30.3)	Erythematokeratodermia variabilis (OMIM 617524)	G12D, R22H, T85P, F137L, F189Y, V37M	Autosomal dominant	Intracellular retention	
				Hyperactive hemichannels	
GJA1 (Cx43)	Keratoderma-hypotrichosis-leukonychia totalis syndrome (OMIM 104100)	G8V	Autosomal dominant	Hyperactive hemichannels	
	Erythematokeratodermia variabilis et progressive (EKVP) (OMIM 617525)	A44V, E227D, P283L, T290N	Autosomal dominant	Hyperactive hemichannels	
	Inflammatory linear verrucous epidermal nevus (ILVEN)	A44V		Hyperactive hemichannels	
	Hypotrichosis with keratosis follicular and hyperostosis	G38E	Autosomal dominant	Hyperactive hemichannels	
	Oculodentodigital dysplasia (ODDD) (OMIM 164200)	K134E	Autosomal dominant	Impaired GJIC	
GJB6 (Cx30)	Clouston's hidrotic ectodermal dysplasia (HED) (OMIM 164200)	G11R, V37E, D50N, A88V	Autosomal dominant	Hyperactive hemichannels	Cx30-A88V mouse model
	Keratitis-ichthyosis-deafness (KID) syndrome	V37E	Autosomal dominant	Hyperactive hemichannels	

### Skin diseases caused by Cx26 gene mutations

Mutations in *GJB2* encoding for Cx26 are associated with several rare syndromic deafness disorders that manifest with skin pathologies, including palmoplantar keratoderma (PPK) with deafness, Vohwinkel’s syndrome (VS), Bart-Pumphrey syndrome (BPS), keratitis-ichthyosis-deafness (KID) syndrome, and hystrix-like-ichthyosis-deafness (HID) syndrome (Srinivas et al., 2018). These diseases arise from dominant mutations in *GJB2*, contrasting non-syndromic deafness associated mutations, which are primarily cause either partial or complete loss of Cx26 function (Mese et al., 2004; 2008; Lee and White, 2009; Posukh et al., 2023). Non-syndromic recessive deafness mutations alter the function of Cx26 only in the inner ear while Cx26 loss in the other tissues including the skin is likely compensated for by other connexins, particularly Cx30 that can form functional heteromeric and heterotypic channels with Cx26 (Scott et al., 2012). On the other hand, *GJB2* mutations result in new features that disrupt the epidermal homeostasis, classifying these disorders as gain-of-function diseases in syndromic deafness cases. To date, at least 14 autosomal dominant mutations have been identified in Cx26 that influence keratinocyte proliferation and differentiation (Richard, 2005; Laird, 2008; Avshalumova et al., 2014; García et al., 2015). Missense mutations primarily affect amino acids located on the N-terminus or the first extracellular loop of the polypeptide. These residues are involved in hemichannel assembly, transport to the cell surface, connexon-connexon interactions, voltage gating, and channel permeability by regulating the opening of cytoplasmic and extracellular pores (Richard et al., 2004; Thomas et al., 2004; Lee and White, 2009; Avshalumova et al., 2014; Dalamón et al., 2016; Srinivas et al., 2018). Gain-of-function mutations are characterized by heteromeric hemichannel formation with wild-type connexin, abnormal oligomerization of mutant Cx26 with other connexins and formation of hyperactive (leaky) hemichannels, depending on the location and the type of mutations (García et al., 2015; Press et al., 2017; Srinivas et al., 2018; Beach et al., 2020).

Palmoplantar keratoderma, characterized by skin thickening in both palms and soles, and autosomal-dominant sensorineural hearing loss are common features of syndromic deafness disorders associated with skin diseases due to Cx26 gene mutations. Depending on the mutation and associated disease, additional skin symptoms can be observed in each syndrome as discussed below (Richard et al., 2004; Scott et al., 2012; Avshalumova et al., 2014; Srinivas et al., 2018). *GJB2* mutations in BPS, VS, and PPK patients reside in similar regions of the protein, resulting in comparable clinical manifestations, classifying them as the first group of syndromic deafness disorders with skin diseases (Lilly et al., 2016). The second group comprises KID and HID syndromes (Srinivas et al., 2018).

#### Palmoplantar keratoderma with deafness

Palmoplantar keratoderma with deafness (PPK) represents the first characterized keratinization disorder linked to *GJB2* mutations (Richard et al., 2004; Lee et al., 2010; Avshalumova et al., 2014; Shuja et al., 2016; Dev et al., 2019). Individuals with PPK exhibit excessive skin thickness on their palms and soles since childhood, resembling the condition in BPS and VS patients. However, they lack any other abnormal ectodermal phenotypes and experience sensorineural hearing loss in a milder form compared to other Cx26 syndromic deafness disorders (Verbov, 1987; Sharland et al., 1992; Richard et al., 1998; Lee and White, 2009; Avshalumova et al., 2014; Has and Technau-Hafsi, 2016; Bedoukian et al., 2021). PPK is associated with several mutations in *GJB2* such as Cx26-DelE42, Cx26-D66H, Cx26-G59A, Cx26-G59R, Cx26-H73R, Cx26-H73Q, Cx26-R75W, Cx26-S183F, Cx26-G130V, and Cx26-M34K (Richard et al., 1998; Heathcote et al., 2000; Kelsell et al., 2000; Rouan et al., 2001; de Zwart-Storm et al., 2008a; de Zwart-Storm et al., 2008b; Iossa et al., 2009; Bedoukian et al., 2021). The specific mechanisms by which these mutations disrupt connexin function vary, ranging from impaired connexin synthesis to altered channel permeability (Rouan et al., 2001; Richard et al., 2002; Thomas et al., 2004; Shuja et al., 2016).

Functional studies in *Xenopus* oocytes expressing Cx26-Del42E, Cx26-D66H, and Cx26-R75W mutations revealed that the mutant proteins were incapable of inducing intercellular coupling when expressed alone. Moreover, when co-expressed with wild-type Cx26, Cx26-Del42E and Cx26-R75W exhibited a profound inhibitory effect on wild-type channel activity while the Cx26-D66H mutation demonstrated a moderately lower inhibitory effect. Moreover, these mutations impaired the Cx43-mediated GJIC in contrast to non-syndromic deafness mutation Cx26-W44C that did not interfere with the Cx43 channel activity. These findings provide the first *in vitro* evidence of the trans-dominant inhibitory effect of PPK-associated Cx26 mutations on wild-type connexins (Rouan et al., 2001)*.* Consistent with these, dye transfer assays demonstrated that Cx26-G59A and Cx26-D66H mutants fail to form functional channels on their own (Thomas et al., 2004) and when co-expressed with either wild-type Cx26 or Cx43, they reduced dye transfer between cells. Notably, Cx26-G59A also exhibited a trans-dominant negative effect on wild-type Cx32, while Cx26-D66H did not affect Cx32 channel function (Marziano et al., 2003; Thomas et al., 2004). Additionally, dye transfer analysis of Cx26-R75W and Cx26-G59A mutations revealed the inhibition of wild-type Cx26 or Cx30 channel activity when co-expressed. The dominant-negative effect of mutations on wild-type Cx26 was demonstrated to be mediated by the co-assembly of mutant and wild-type proteins into heteromeric channels as shown by their physical interaction through co-localization and co-immunoprecipitation experiments (Marziano et al., 2003; Yum et al., 2010; Zhang et al., 2011).

Similar to other PPK mutations, Cx26-S183F and Cx26-H73R mutations failed to form hemichannels or GJ channels when expressed alone (Shuja et al., 2016). Consistent with previous findings, co-expression with wild-type Cx43 resulted in a trans-dominant inhibition of Cx43 GJ channel function without affecting Cx43 protein levels. Moreover, mutant Cx26 proteins formed heteromeric connexons with wild-type Cx43, which had increased Cx43 hemichannel activity in cells (Shuja et al., 2016). Cx26-S183F mutation caused retention of proteins within intracellular compartments while some of them formed GJ plaques on the plasma membrane. When co-expressed with wild-type Cx26, Cx30 or Cx43, Cx26-S183F mildly inhibited Cx26 channels and exerted a trans-dominant inhibitory effect on Cx30 channel function (Press et al., 2017). Finally, a recently identified PPK mutation, Cx26-M34K, was shown to be retained in the ER and failed to form GJ channels on the plasma membrane (Bedoukian et al., 2021). Mutant proteins also prevented the delivery of the wild-type Cx26 to the plasma membrane when co-expressed, exerting a dominant-negative effect on wild-type channels.

Collectively, these studies have elucidated a common gain-of-function mechanism for *GJB2* mutations associated with PPK. The mutant proteins inhibited GJ channel activity mediated by wild-type connexins, including Cx26, Cx30, Cx32, and Cx43. The dominant suppression of wild-type connexins by mutant Cx26 proteins can lead to a reduction in the number of functional GJ channel types in the epidermis, altering the GJIC between cells that can contribute to its pathogenesis.

#### Vohwinkel syndrome

Vohwinkel syndrome (VS) was first described in 1929. It is a rare genetic disorder characterized by palmoplantar hyperkeratosis, sensorineural hearing loss, and specific skin manifestations (Vohwinkel, 1929; Lee and White, 2009). Patients typically develop honeycomb-like calluses on their palms and soles during infancy or early childhood. These lesions are often accompanied by constriction bands known as pseudoainhum, which can restrict blood flow and potentially lead to autoamputation of the affected digit. Moreover, VS patients may exhibit thickened, starfish-shaped patches of skin on top of their fingers and knees and the severity of sensorineural hearing loss can range from mild to moderate (Maestrini et al., 1999; Snoeckx et al., 2005; Guerra et al., 2018; Xie et al., 2019; Bedoukian et al., 2021). The restriction of skin pathology to the palms and soles is also observed in individuals with BPS and PPK. However, these conditions lack the constriction bands and starfish-shaped keratoses characteristics of VS (Lilly et al., 2016; Dev et al., 2019; Bedoukian et al., 2021). In addition, ichthyosis, or dry, scaly skin, and scarring alopecia, or hair loss, found in individuals with KID and HID syndromes are not observed in VS, further aiding in the distinction among these disorders (Dev et al., 2019; Bedoukian et al., 2021). The classic form of VS is caused by mutations in the *GJB2* gene, while a variant form of VS, characterized by ichthyosis and VS without hearing loss, is caused by a single-base pair insertion mutation in the *LOR* gene, encoding for loricrin (Govender and Pillay, 2023). Both “classical” form and the variant form of VS are characterized by pseudoainhum (Ling et al., 2023).

Vohwinkel syndrome (VS) has been attributed to distinct mutations in Cx26: G59S, Y65H, D66H, and G130V (Maestrini et al., 1999; Alexandrino et al., 2005; Snoeckx et al., 2005; Bondeson et al., 2006; Iossa et al., 2009; de Zwart-Storm et al., 2011). The Cx26-G59S mutation, associated with mutilating keratoderma, ichthyosis, and congenital deafness, likely disrupts Cx26 transport and hemichannel permeability, potentially contributing to the pathogenesis of VS as alterations in the first extracellular domain have been shown to affect Cx26 transport and hemichannel permeability. (Bondeson et al., 2006; de Zwart-Storm et al., 2011; Qiu et al., 2012; Xie et al., 2019). Furthermore, individuals carrying the Cx26-G59S mutation exhibit an increased susceptibility to skin cancer (Bondeson et al., 2006; Qiu et al., 2012). The Cx26-Y65H and Cx26-D66H mutations, both located within the first extracellular domain of Cx26, induced intracellular aggregate formation and had residual GJ plaques on the plasma membrane (Maestrini et al., 1999; Bakirtzis et al., 2003; Qiu et al., 2012). Moreover, these mutations impaired GJIC between cells, as demonstrated in parachute assays (Thomas et al., 2004; de Zwart-Storm et al., 2011; Qiu et al., 2012). In a transgenic VS mouse model, the expression of Cx26-D66H mutation in suprabasal keratinocytes under the K10 promoter resulted in the development of epidermal scaling, hyperkeratosis, and constriction bands, particularly in the tail, leading to auto-amputation (Bakirtzis et al., 2003). Similar to *in vitro* observations, both wild-type Cx26 and Cx30 was accumulated within suprabasal keratinocytes, leading to the disruption of epidermal GJIC and possibly premature terminal differentiation of cells.

Cx26-G130V, located in the second intracellular domain, was also associated with VS, in addition to PPK (Snoeckx et al., 2005; Iossa et al., 2009). HaCaT cells expressing Cx26-G130V or Cx26-D66H had reduced proliferation and migration rate in addition to decreased transforming growth factor β1 (TGF-β1) expression compared to control cells (Ling et al., 2023). Treatment of cells with TGF-β1 inhibitor decreased proliferation in mutant cells while addition of TGF-β1 rescued the proliferation and migration phenotype, which might indicate the potential of low dose of TGF-β1 for VS treatment.

Overall, functional characterization of mutations suggested the loss of GJIC between cells as a common underlying mechanism for epidermal phenotypes of VS cases. In addition, their intracellular retention can interfere with the expression and function of other molecules involved in keratinocyte proliferation or differentiation such as TGF-β1.

#### Bart-Pumphrey syndrome

Bart-Pumphrey syndrome (BPS), also known as Schwann syndrome, was first reported in a six-generation family by Bart and Pumphrey in 1967 (Bart and Pumphrey, 1967). Prior to Bart and Pumphrey, Schwann had documented a child exhibiting all BPS symptoms; however, no other affected individuals were identified within his family (Schwann, 1963; Gonul et al., 2012). The syndrome is characterized by congenital deafness, knuckle pads, and leukonychia. Additionally, palmoplantar hyperkeratosis has been observed in some patients (Alexandrino et al., 2005; Gonul et al., 2012; Guerra et al., 2018). The abnormal skin phenotypes become more pronounced in childhood, whereas hearing loss is present from birth. Despite these challenges, individuals with BPS generally have a normal life expectancy (Bart and Pumphrey, 1967; Richard et al., 2004; Avshalumova et al., 2014; Al-Hamdi et al., 2020; Al-Hamdi et al., 2020).

Two distinct *GJB2* mutations, Cx26-N54K and Cx26-G59S, have been identified in BPS. They reside in the highly conserved first extracellular loop, which plays critical roles in hydrogen bond-mediated hemichannel docking, forming functional GJ channels, and voltage gating (Richard et al., 2004; Alexandrino et al., 2005; Beach et al., 2020). The Cx26-N54K mutation, which replaces a highly conserved asparagine with lysine, result into intracellular retention of the protein (Richard et al., 2004; Press et al., 2017). Immunohistochemical staining of palmar and knuckle epidermis from a BPS patient carrying Cx26-N54K exhibited reduced Cx26 expression, while a more widespread expression of Cx30 was observed in lesional skin, possibly as a compensatory mechanism to overcome the abnormal Cx26 function (Richard et al., 2004). Moreover, HeLa cells carrying the Cx26-N54K formed significantly fewer gap junctional plaques compared to wild-type cells. When it was co-expressed with wild-type Cx26, even though more GJ plaques were observed compared to mutant-alone cells, the channels were unable to transfer fluorescent dyes, demonstrating the dominant-negative effect of the mutation on wild-type channel function. Furthermore, the mutation caused the retention of Cx30 in intracellular compartments, reducing the formation of Cx30 GJ plaques on the plasma membrane and decreased dye transfer among cells, suggesting the trans-dominant effect of the mutation on Cx30 channel function. Similarly, Cx26-N54K exhibited a trans-dominant effect on Cx43, reducing the transfer of fluorescent dyes between rat epidermal keratinocytes co-expressing Cx26-N54K and Cx43 (Press et al., 2017; Beach et al., 2020).

The Cx26-G59S mutation, which replaces glycine with serine at codon 59 in the first extracellular loop, can affect transport and hemichannel activity. This mutation is also observed in VS cases (Xie et al., 2019). While BPS patients share some clinical symptoms with VS, they can be distinguished by the presence of knuckle pads and leukonychia (Maestrini et al., 1999; Alexandrino et al., 2005; Xie et al., 2019). Although the effect of Cx26-G59S mutation on connexin biogenesis and channel function remain unclear, studies on Cx26-N54K suggest a dominant-negative effect on wild-type connexins, which can possibly contribute to BPS pathogenesis by altering GJIC between keratinocytes.

#### Keratitis-ichthyosis-deafness and hystrix-like ichthyosis syndrome

Keratitis-ichthyosis-deafness (KID) and hystrix-like ichthyosis with deafness (HID) syndromes are both rare autosomal dominant disorders characterized by sensorineural hearing loss, thickening of the stratum corneum (hyperkeratosis) and keratitis. Scarring alopecia and hair loss due to follicular hyperkeratosis may occur as well. HID syndrome is characterized by erythroderma and hystrix-like ichthyosis, characterized by thick and warty skin. Moreover, erythroderma typically develops shortly after birth. After 1 year, hyperkeratosis intensifies, forming rough, cobblestone-like patches that spread across the entire body (van Geel et al., 2002; van Steensel et al., 2002; Todt et al., 2006). Patients with KID syndrome develop erythrokeratoderma from birth and severe palmoplantar hyperkeratosis that extends beyond the palms and soles (Caceres-Rios et al., 1996; Lee and White, 2009; Markova et al., 2016; Xu et al., 2022). The palms and soles of HID patients exhibit milder hyperkeratosis compared to KID patients, and keratitis is less severe (Todt et al., 2006). In addition, HID syndrome distinguishes from KID syndrome by the severity and timing of the abnormal skin manifestations so HID syndrome is considered a phenotypic variant of KID syndrome (Lilly et al., 2016).

KID syndrome arises from missense mutations in the *GJB2* gene (Richards et al., 2002; van Steensel et al., 2002; Mazereeuw-Hautier, 2007). The most common mutation, accounting for nearly 80% of cases, is a substitution of a highly conserved aspartic acid at position 50 with asparagine (D50N) (Nyquist et al., 2007). This mutation disrupts voltage gating, a critical process for regulating ion flow through Cx26 channels. Individuals with the Cx26-D50N mutation often experience severe sensorineural deafness, keratitis, and vision problems (Rubin et al., 1992; Mazereeuw-Hautier, 2007). Despite the severity of their symptoms, patients with the Cx26-D50N mutation can live well into adulthood. However, they carry an elevated risk of developing squamous cell carcinoma (Grob et al., 1987; Lee et al., 2009; Sellitto et al., 2021). In addition, D50N was also identified in a patient with HID syndrome (Todt et al., 2006). The underlying mechanism for this increased risk is not fully understood, but it is thought to be related to the disruption of GJIC, which can lead to uncontrolled cell growth.

The severity of the skin phenotypes varies greatly among KID syndrome patients. Patients with Cx26-D50N or Cx26-S17F, manifest milder phenotypes (Lilly et al., 2016). On the other hand, rarely observed Cx26-G45E and Cx26-A88V mutations can have more severe consequences, including lethality, particularly in infants during their first year of life (Jonard et al., 2008; Mese et al., 2011; Mhaske et al., 2013). Individuals bearing these mutations typically exhibit congenital hearing loss and severe skin infections that can progress into septicemia and ultimately death (Jonard et al., 2008; Koppelhus et al., 2011; Mhaske et al., 2013). Therefore, the characterization of each mutation would be crucial to understanding the mechanisms underlying different phenotypes in patients.

Cx26-D50N or Cx26-G11E mutations disrupted trafficking of Cx26 protein, leading to the accumulation of the protein within cells and the disorganization of its distribution on the plasma membrane. This then prevented the formation of structured hemichannels in normal human keratinocyte (NHEK) cells. The Cx26-G11E mutation additionally exhibited a predominant cytoplasmic perinuclear localization whereas the wild-type cells formed GJ channels on the plasma membrane (Terrinoni et al., 2010). Moreover, NHEK cells harboring Cx26-D50N or Cx26-G11E displayed elevated cytoplasmic Ca^2+^ levels compared to wild-type Cx26 cells. Furthermore, both mutations induced necrosis, with a more pronounced effect observed in Cx26-G11E-containing cells. These findings suggest that the formation of hyperactive hemichannels due to Cx26-D50N and Cx26-G11E mutations can lead to dysregulated Ca^2+^ homeostasis, increasing intracellular Ca^2+^ levels and promoting necrotic cell death (Terrinoni et al., 2010).

Similar to Cx26-D50N and Cx26-G11E, Cx26-G12R and Cx26-N14K mutations resulted in increased hemichannel activity compared to wild-type Cx26-expressing cells (Lee et al., 2009). This increased hemichannel activity was also linked to cell death. Interestingly, the cell death can be reversed by increasing the extracellular Ca^2+^ concentration. However, the response to increased Ca^2+^ concentration varied between the mutations. As the extracellular Ca^2+^ concentration was elevated, the reduction in hemichannel current was more pronounced in Cx26-G12R cells compared to Cx26-D50N cells. This suggests that these mutations affect channel function in distinct ways (Lee et al., 2009). On the other hand, another mutation, Cx26-S17F located on the N-terminus of the protein, resulted in the complete loss of both gap junctional coupling and hemichannel activity. Unlike the other mutations, Cx26-S17F did not induce cell death (Lee et al., 2009). The failure of mutants to form functional hemichannels or GJ channels altered the GJIC between cells, which might disrupt epidermal proliferation and differentiation (Schütz et al., 2011). Despite the complete loss of gap junctional conductance and hemichannel activity, dye-coupling experiments in HeLa cells demonstrated that the mutant proteins could still be transported to the membrane and assembled into GJ plaques (Richard et al., 2002; Schütz et al., 2011). In a mouse model harboring the Cx26-S17F mutation, homozygous mutants were not viable, while heterozygous animals exhibited epidermal hyperplasia on their tail and footpads. Moreover, adult mice developed hearing impairment, possibly due to altered ionic homeostasis in the inner ear (Schütz et al., 2011). In addition, another mouse model expressing Cx26-S17F under the control of cytokeratin 14 promoter in keratinocytes had abnormal keratinocyte proliferation and differentiation in footpad (Press et al., 2017). Moreover, Cx26, Cx30, and Cx43 had wider distribution than control animals where they were observed in most keratinocytes in the epidermis. Unlike other KID syndrome mutations leading to increased hemichannel activity, the Cx26-S17F mutation disrupted intercellular coupling even though the protein was properly transported within the cell (Richard et al., 2002; Schütz et al., 2011; Garcia et al., 2015). Interestingly, the co-expression of Cx26-S17F with wild-type Cx26 or Cx43 resulted in the formation of hyperactive hemichannels, which elevated intracellular Ca^2+^ levels and increased ATP release (Garcia et al., 2015).

One of the most severe mutations, Cx26-G45E caused the formation of channels on the plasma membrane with significantly higher cell membrane currents compared to wild-type Cx26, producing hyperactive hemichannels and GJ channels (Stong et al., 2006). The elevated membrane currents and constant opening of hemichannels eventually resulted in cell lysis and death. Notably, increasing extracellular Ca^2+^ levels in a dose-dependent manner could mitigate the severe phenotype by inducing the closure of hyperactive hemichannels (Stong et al., 2006; Gerido et al., 2007; Jonard et al., 2008; Mese et al., 2011; Sanchez and Verselis, 2014; Taki et al., 2018). An inducible transgenic mouse model expressing the Cx26-G45E mutation specifically in keratinocytes exhibited reduced viability, hyperkeratosis, skin scaling, and scarring alopecia, hence mimicking the pathological features of KID syndrome. Moreover, hemichannel activity in transgenic keratinocytes were elevated as shown by increased membrane current, further validating the contribution of increased hemichannel activity in the pathogenicity of the Cx26-G45E mutation (Mese et al., 2011). Similar to Cx26-G45E, the Cx26-A40V and Cx26-A88V variants formed aberrant hemichannels, resulting in hyperactive cell membranes (Mhaske et al., 2013; Sanchez and Verselis, 2014; Aypek et al., 2016). In addition, Cx26-A40V hemichannels exhibited substantial impairment in closing and nearly doubled hemichannel activity due to acidification (Sanchez and Verselis, 2014). Moreover, Cx26-A40V, Cx26-G45E, and Cx26-D50N showed varying degrees of closure in the presence of extracellular Ca^+2^ and only Cx26-A40V hemichannels demonstrated reduced sensitive to pH (Gerido et al., 2007; Lee et al., 2009; Sanchez and Verselis, 2014; Sanchez and Verselis, 2014).

Functional characterization of Cx26-KID syndrome mutations revealed that mutations caused the formation of hyperactive hemichannels on the plasma membrane either alone or when co-expressed with Cx26 or Cx43 (Taki et al., 2018). These hyperactive hemichannels can lead to uncontrolled molecular exchange between the cytosol and the extracellular environment, which can not only promote cell death but also influence the behavior of surrounding cells, interfering with the proliferation and differentiation of keratinocytes. Thus, the dysregulated molecular exchange across the plasma membrane through hyperactive hemichannels may be a common feature, underlying the epidermal phenotypes in Cx26 KID syndrome mutations (Kelsell et al., 2001; Stong et al., 2006; Gerido et al., 2007; Jonard et al., 2008; Sanchez and Verselis, 2014; Taki et al., 2018).

### Skin diseases caused by Cx31 and Cx30.3 gene mutations

Erythematokeratodermia variabilis et progressive (EKVP) is the first reported skin disease linked to mutations in connexins (Richard et al., 1998; Richard, 2005) and mutations in *GJB3, GJB4,* and *GJA1*, encoding Cx31, Cx30.3 and Cx43, respectively cause EKVP (Duchatelet and Hovnanian, 2015; Ishida-Yamamoto, 2016; Laird and Lampe, 2022). EKVP is characterized by fixed hyperkeratotic plaques and transient erythematous patches and is mostly inherited in an autosomal dominant inheritance but homozygous *GJB3* mutations also lead to EKVP in some rare cases, suggesting an autosomal recessive inheritance (Gottfried et al., 2002; He et al., 2005; Terrinoni et al., 2010; Zhang et al., 2022).

The analysis of Cx31-EKVP mutations revealed their distinct effects on connexin and GJ biogenesis and function for recessive and dominant mutations. Recessive mutations, such as Cx31-L34P and Cx31-V30I, caused the retention of proteins in the cytoplasm, which reduced the formation of GJ channels on the plasma membrane (Gottfried et al., 2002; He et al., 2005; Fuchs-Telem et al., 2011; Tang et al., 2015; Laird and Lampe, 2022). In contrast, dominant mutations caused more severe cellular phenotypes through various molecular mechanisms, leading to necrotic cell death (Diestel et al., 2002; He et al., 2005; Tattersall et al., 2009; Chi et al., 2012; Easton et al., 2019). The expression of the Cx31-G12R mutation in HeLa cells resulted in increased GJIC between cells, which could potentially play a role in cell death by altering the types and/or the amount of molecules exchanged between cells (Diestel et al., 2002). In contrast, Cx31-G12D, Cx31-R42P, and Cx31-C86S caused endoplasmic reticulum (ER) stress and activated unfolded protein responses in cells, which was suggested to be the primary cause of Cx31 mutation-dependent cell deaths. As cell death was not completely prevented in the presence of high extracellular Ca^2+^ levels, constitutive active hemichannels were not considered to be the main contributor to cell death (Tattersall et al., 2009). Induction of ER stress was suggested to lead to abnormal keratinocyte proliferation and/or differentiation in patients with these mutations (Tattersall et al., 2009). Furthermore, Cx31-R42P mutation caused the formation of constitutively active hemichannels, increased ER stress, and produced reactive oxygen species (ROS), which could eventually induce necrotic cell death (Chi et al., 2012). Additionally, cells exhibited elevated ATP release into the extracellular space. Inhibition of hemichannels or reduction of ROS prevented cell death and ATP release, indicating the role of hyperactive hemichannels in EKVP pathogenesis.

During the generation of a mouse model for EKVP, the Cx31-F137L mutation reduced GJIC between mouse embryonic stem cells (Schnichels et al., 2007). Moreover, Cx31-F137L heterozygote adult mice exhibited a shortened healing process for tail incision wounds, which was similar to mice with reduced Cx43 expression. This similarity and the reduction of GJIC between mouse embryonic stem cells suggested an interaction between Cx31 and Cx43 and a dominant negative effect of Cx31-F137L mutation on Cx43-dependent GJIC. In addition, in *Drosophila*, the expression of the Cx31-F137L mutation resulted in the loss of pigmentation and degeneration in ommatidia that was rescued by co-expression of BiP or Hsp70 chaperon proteins, indicating the involvement of the unfolded protein response (Tang et al., 2015). Moreover, overexpression of Cx31-WT in mouse skin led to hyperproliferation and abnormal differentiation, mimicking the skin phenotypes of EKVP patients. Notably, c-Fos and JunB, key components of the epidermal keratinocyte survival and differentiation regulator AP-1 transcription factor complex, were elevated in Cx31-WT overexpressing mouse skin and skin lesions of EKVP patients compared to controls. Furthermore, treatment of Cx31-WT mouse skin with an AP-1 inhibitor suppressed the EKVP-like phenotypes, further supporting the role of dysregulated AP-1 in EKVP pathology (Tang et al., 2015).

A recently identified Cx31-G45E mutation was also shown to accumulate in the ER and disrupted cellular structures such as the ER and microtubule network, without triggering ER stress (Easton et al., 2019). Despite accumulating in the ER, Cx31-G45E could still interact with Cx31-WT proteins when co-expressed and partially co-localized at the plasma membrane. Interestingly, Cx31-G45E extensively co-localized with Cx43 and significantly hindered its trafficking to the plasma membrane as well, disrupting Cx43 function. Consequently, the dominant-negative effect of Cx31-G45E mutation on wild-type connexins likely contributes to the skin phenotypes observed in EKVP by altering the GJIC between cells. Collectively, Cx31 EKVP mutations cause diverse changes in cellular processes, such as ER stress, disruption of cellular structures, altered intercellular communication, and cell death, ultimately interfering with keratinocyte proliferation and differentiation.

Several mutations in *GJB4* including Cx30.3-G12D, Cx30.3-R22H, Cx30.3-T85P, Cx30.3-F137L, and Cx30.3-F189Y have been also linked to EKVP, highlighting the role of Cx30.3 in epidermal differentiation (Macari et al., 2000; Richard et al., 2003). Recently, a novel Cx30.3-V37M mutation was identified in a sporadic and late-onset EKVP case. This mutation significantly reduced Cx30.3 expression in the patient’s epidermis, and consistent with this, the mutant protein displayed reduced expression and a more diffuse distribution compared to the membrane-localized wild-type Cx30.3 in HeLa cells (Zhang et al., 2022). Similarly, Cx30.3-G12D, Cx30.3-T85P, and Cx30.3-F189Y mutant proteins remained within the ER and exhibited impaired transport to the plasma membrane in rat epidermal keratinocytes (Lucaciu et al., 2023). Interestingly, these mutant protein-expressing cells also exhibited increased propidium iodide uptake, potentially indicating the presence of active hemichannels. Notably, co-expression of either wild-type Cx30.3 or other skin connexins (Cx26, Cx30, and Cx43) with Cx30.3 mutants facilitated the incorporation of mutant proteins into GJ at the plasma membrane. This finding suggests that upregulation of wild-type connexins in the epidermis may hold therapeutic potential for Cx30.3-related EKVP (Lucaciu et al., 2023).

The observed association between Cx30.3, Cx31, and Cx43 expression patterns in skin (White and Bruzzone, 1996; Plantard et al., 2003) may explain the overlapping clinical symptoms frequently observed in EKVP patients with mutations in these genes (Ishida-Yamamoto, 2016). A common feature of many EKVP-causing mutations is their impact on the trafficking of mutant connexins. These mutations prevent the mutant proteins from reaching the cell membrane, hindering the formation of functional GJ channels and hence altering cellular communication between keratinocytes.

### Skin diseases caused by Cx43 gene mutations

Cx43, encoded by the *GJA1* gene, is a crucial component of the epidermal, dermal, and hypodermal layers of the skin. In addition to the epidermis, Cx43 is also found in other skin cells including fibroblasts and melanocytes (Zhang and Cui, 2017). *GJA1* mutations have been shown to lead to various hereditary skin disorders (Lilly et al., 2016; Qiu et al., 2022). One such example is the Cx43-G8V mutation, linked to keratoderma-hypotrichosis-leukonychia totalis syndrome (KHLS), which is characterized by severe hyperkeratosis, congenital alopecia, and leukonychia totalis (Wang et al., 2015). *In vitro* studies using HEK293 cells expressing the Cx43-G8V mutation revealed that mutant cells could still form GJs similar to those formed by wild-type Cx43. However, mutant hemichannels displayed increased cell membrane current, suggesting enhanced activity compared to wild-type Cx43. These hyperactive hemichannels facilitated Ca^2+^ influx at resting potential, raising cytoplasmic Ca^2+^ levels and ultimately triggering keratinocyte apoptosis, which likely contribute to hyperkeratosis in KHLS (Wang et al., 2015; Srinivas et al., 2019).

Other Cx43 mutations such as Cx43-A44V and Cx43-E227D have been identified as causative factors for EKVP (Boyden et al., 2015). As explained before, EKVP is characterized by widespread or localized hyperkeratosis and approximately half of EKVP cases develop palmoplantar keratoderma as well. Additionally, patients with *GJA1*-related EKVP often exhibit white crescents at the base of their nails and periorificial darkening. Cx43-EKVP mutations caused the mislocalization of the protein where they were accumulated in the Golgi apparatus (Boyden et al., 2015; Ishida-Yamamoto, 2016; Srinivas et al., 2019).

Functional studies in *Xenopus* oocytes or transiently transfected HeLa cells carrying Cx43-G8V, Cx43-A44V, and Cx43-E227D mutations, demonstrated that all mutations retained the ability to form functional GJ channels, similar to wild-type Cx43 (Srinivas et al., 2019). Interestingly, they did not alter voltage gating, unitary channel conductance, protein expression levels, or cellular localization. However, they all caused the formation of hyperactive hemichannels, exhibiting significantly increased membrane currents, in contrast to wild-type Cx43 channels. Thus, this enhanced hemichannel activity appears to be a unifying mechanism underlying the pathophysiology of Cx43-linked skin disorders (Srinivas et al., 2019). Further expanding on the spectrum of Cx43 mutations involved in EKVP, two *de novo* missense mutations, i.e., Cx43-P283L and Cx43-T290N, have been identified in two unrelated EKVP patients. Interestingly, both patients carried the Cx43-P283L mutation, while one patient harbored the Cx43-T290N mutation as well. Immunohistochemistry and immunofluorescence analysis revealed that Cx43 protein was observed both on the plasma membrane and in the cytoplasm of cells in the stratum corneum and granular layer, suggesting that Cx43-P283L and Cx43-T290N mutations caused Cx43 mislocalization to intracellular compartments. Notably, the patient with both mutations exhibited a more severe phenotype and a higher degree of cytoplasmic accumulation (Li et al., 2019). Additionally, the Cx43-A44V mutation has also been associated with inflammatory linear verrucous epidermal nevus (ILVEN), a skin condition characterized by pruritic, erythematous, and hyperkeratotic papules arranged linearly along Blaschko’s lines (Umegaki-Arao et al., 2017; Cocozzelli and White, 2019).

Another reported case of a hyperactive hemichannel is associated with a new *GJA1* mutation, Cx43-G38E. This mutation was linked to hypotrichosis with keratosis follicular and hyperostosis, a severe condition affecting both the skin and skeleton (Bursztejn et al., 2019). When expressed in HeLa cells, Cx43-G38E formed GJ plaques similar to those observed in wild-type cells (Crouthamel et al., 2023). Furthermore, studies in *Xenopus* oocytes showed that Cx43-G38E formed functional GJ channels with altered voltage gating properties. However, the key finding is that the Cx43-G38E mutation also created hyperactive hemichannels in oocytes, suggesting the role of increased hemichannel activity in this disorder.

Oculodentodigital dysplasia (ODDD), a rare genetic disorder inherited in an autosomal dominant pattern, is also caused by *GJA1* mutations. It is characterized by abnormalities in facial bones (eyes, nose, and teeth), limb malformations, fused fingers or toes, and defects affecting the eyes, the skin, hair, and nails. Unlike other connexin-associated syndromes, ODDD patients typically experience conductive hearing loss rather than sensorineural hearing loss (Gillespie, 1964; Paznekas et al., 2003; Avshalumova et al., 2014; Kelly et al., 2016; Lilly et al., 2016; Qiu et al., 2022). Skin manifestations are less common and typically mild in ODDD, including hair loss and brittle nails. Palmoplantar keratoderma is exceptionally rare in ODDD (van Steensel et al., 2005; Boyden et al., 2015). A sporadic case of ODDD characterized by hyperkeratosis on the palms and soles was associated with the Cx43-K134E mutation (Paznekas et al., 2003; Shibayama et al., 2005). Functional studies in N2A cells revealed that, although the mutant Cx43-K134E protein could still form GJ plaques, the unitary conductance of these mutant channels was reduced (Seki et al., 2004). Moreover, dual patch-clamp recordings further confirmed that Cx43-K134E channels mediated electrical coupling between paired cells, demonstrating their functionality. However, there was a significant decrease in macroscopic junctional conductance between cells carrying mutant proteins compared to wild-type Cx43 (Shibayama et al., 2005). This impaired GJIC might be important in the development of the skin phenotype in ODDD patients, which need to be further validated by characterizing other mutations. KHLS, EKVP, and ILVEN stand apart from ODDD as non-syndromic skin-limited diseases caused by Cx43 mutations. Notably, they lack all the diagnostic features associated with ODDD, further highlighting their distinct clinical profiles (Boyden et al., 2015).

### Skin diseases caused by Cx30 gene mutations

Hidrotic ectodermal dysplasia (HED), also known as Clouston syndrome, is a rare autosomal dominant genetic disorder, which is characterized by alopecia, nail dystrophy and palmoplantar hyperkeratosis. These common features can sometimes be accompanied by additional phenotypes such as the hyperpigmentation of the skin and developmental defects including polydactyly or syndactyly and intellectual disability (Cammarata-Scalisi et al., 2019). Mutations in the *GJB6* gene, encoding Cx30, are responsible for Clouston syndrome. Several specific mutations have been identified, including Cx30-G11R, Cx30-V37E, Cx30-D50N, and Cx30-A88V (Lamartine et al., 2000; Common et al., 2002; Fujimoto et al., 2013; Bosen et al., 2014; Lilly et al., 2016). Interestingly, the Cx30-V37E mutation can also lead to KID syndrome with severe keratitis and deafness, highlighting the heterogeneity of KID syndrome (Jan et al., 2004).

Expression of Cx30-G11R and Cx30-A88V mutations resulted in their cytoplasmic accumulation in HeLa cells (Essenfelder et al., 2004; Berger et al., 2014). However, co-expression with wild-type Cx30 rescued their trafficking to the plasma membrane, indicating functional GJ formation (Essenfelder et al., 2004). In addition, the mutant proteins formed hyperactive hemichannels, leading to ATP release into the extracellular environment (Essenfelder et al., 2004). Interestingly, Cx30-A88V exhibited partial functionality and dominant-negative effect on wild-type connexins, failing to be rescued by co-expression of Cx30, Cx26, or Cx43 (Berger et al., 2014; Bosen et al., 2014). Moreover, Cx30-G11R and Cx30-A88V inhibited cell growth and increased apoptosis markers in HaCaT cells, suggesting the involvement of cell death in pathogenesis of Clouston syndrome (Lu et al., 2018).

A mouse model expressing Cx30-A88V under the endogenous Cx30 promoter recapitulated key Clouston syndrome features, including mild palmoplantar hyperkeratosis, enlarged sebaceous glands, and deafness (Bosen et al., 2014). Similar to *in vitro* studies, the Cx30-A88V mutant proteins incorporated into gap junctional plaques, suggesting some degree of functionality. Notably, the mutation had minimal impact on Cx26 expression (Bosen et al., 2014). In addition, hyperactive hemichannel activity was confirmed by administrating a monoclonal antibody targeting Cx26 hemichannels, abEC1.1. This antibody reduced the numbers of sebocytes and proliferating keratinocytes compared to controls (Kuang et al., 2020). Notably, it also blocked Ca^2+^ influx and ATP release in primary mouse keratinocytes and HaCaT cells with Cx30-A88V, indicating disruption of epidermal differentiation by increasing extracellular ATP. ATP plays a crucial role in keratinocyte differentiation and proliferation, which can be mediated through purinergic receptor activation and subsequent Ca^2+^ release (Denda et al., 2002; Essenfelder et al., 2004). Therefore, the elevated extracellular ATP released by hyperactive hemichannels likely acts as a paracrine signal, disrupting epidermal differentiation and potentially contributing to Clouston syndrome. Finally, the direct regulation of the *GJB6* gene by the transcription factor p63, particularly by the ΔNp63α isoform known to be a key regulator of epidermal differentiation (Fujimoto et al., 2013; Eyermann et al., 2024), highlights the fundamental link between Cx30 activity and the maintenance of a healthy epidermis.

## Connexin dysregulation in other skin conditions

While mutations in connexin genes can directly cause skin diseases, their expression is also dysregulated in other skin conditions, including psoriasis, pressure ulcers, wound healing issues, and even skin cancers (Garcia-Vega et al., 2021). Psoriasis, an inflammatory skin disorder characterized by thickened and scaly lesions, serves as a major example. Studies revealed a dramatic upregulation of Cx26 in psoriatic lesions, suggesting it as a marker for psoriasis (Rivas et al., 1997; Labarthe et al., 1998; Lucke et al., 1999; Sun et al., 2010; Li et al., 2014; Martin et al., 2014; Yao et al., 2015; Garcia-Vega et al., 2021). Increased Cx26 expression by stimulation of pro-inflammatory responses promoted the release of inflammatory molecules such as ATP and IL-6 into the extracellular environment, indicating the contribution of elevated hemichannels in this process (Garcia-Vega et al., 2019). Additionally, mice engineered to overexpress Cx26 in keratinocytes exhibited psoriasis-like phenotypes, supporting the link between Cx26 and impaired barrier function (Djalilian et al., 2006).

Cx43 presents a more complex picture in psoriasis. Its levels were slightly elevated in some studies (Labarthe et al., 1998), while others reported a decrease in psoriatic lesions (Liang et al., 2019). This discrepancy might be explained by recent findings suggesting that Cx43 in psoriatic tissues underwent post-translational modifications, especially phosphorylation that might interfere with Cx43 biogenesis rather than increased mRNA production (O’Shaughnessy et al., 2021). Interestingly, IL-22, a cytokine involved in psoriatic keratinocyte hyperproliferation, seems to downregulate Cx43 expression, further hindering GJIC (Liang et al., 2019).

### Connexins in chronic non-healing wound healings

Connexins are also implicated in the complicated processes of wound healing. Their dynamic expression patterns orchestrate cellular events spanning inflammation, migration, and tissue remodeling, dictating the temporal progression of repair (Brandner et al., 2004; Garcia-Vega et al., 2021). In acute wounds, precisely timed events take place. Cx43, normally abundant in the healthy epidermis, undergoes downregulation at wound edges, facilitating keratinocyte migration and accelerating closure (Kretz et al., 2003; Scott et al., 2012). This phenomenon aligns with findings that Cx43 inhibition or mimetic peptide application promoted faster healing and reduced inflammation (Qiu et al., 2003; Ghatnekar et al., 2009; Wright et al., 2009). Concurrently, Cx26 and Cx30, normally expressed at low levels, become upregulated surrounding the wound, suggesting their involvement in early repair phases (Kretz et al., 2003; Scott et al., 2012). However, this delicately balanced expression profile can become dysregulated in chronic wounds. Persistent inflammation and delayed healing are often associated with an aberrant elevation of Cx26, Cx30, and Cx43, disrupting intercellular communication and hindering repair (Brandner et al., 2004; Wang et al., 2007; Mendoza-Naranjo et al., 2012; Sutcliffe et al., 2015; Kwek et al., 2023).

Chronic wounds, unlike acute wounds, fail to progress through the ordered sequence of repair process. This altered healing process manifests in a distinct gene expression profile, including connexins. Notably, in chronic non-healing wounds such as venous leg ulcers, diabetic foot ulcers, and pressure ulcers, epidermal and dermal Cx26, Cx30, and Cx43 exhibited upregulation at the wound edge (Brandner et al., 2004; Wang et al., 2007; Mendoza-Naranjo et al., 2012; Sutcliffe et al., 2015; Kwek et al., 2023). This aberrant expression pattern was further associated with delayed wound closure, particularly in the case of diabetic foot ulcers (Brandner et al., 2004; Wang et al., 2007; Mendoza-Naranjo et al., 2012). Despite this association, targeting Cx43 with mimetic peptides such as Gap27 in wound models has yielded mixed results. While Gap27 demonstrated efficacy in non-diabetic models (Wright et al., 2012), its effect on migration and proliferation in diabetic cells appeared limited, suggesting a distinct role for Cx43 in different wound healing contexts (Pollok et al., 2011). Despite this complexity, Cx43 remains a promising target for therapeutic intervention in chronic wounds. Mimetic peptides such as Gap27 and αCT1 have shown success in improving wound closure rates in both chronic and acute wounds. They were implicated in enhanced cell migration, altered extracellular matrix deposition, and improved cell-to-matrix adhesion (Ghatnekar et al., 2009; Wright et al., 2009; Wright et al., 2012; Moore et al., 2013; Moore et al., 2014; Laird and Lampe, 2018).

In conclusion, connexins play a crucial role in the intricate processes of wound healing, with each stage characterized by a distinct connexin environment (Solan and Lampe, 2015; Garcia-Vega et al., 2021). Understanding the dysregulation of this environment in chronic wounds and developing targeted therapies that modulate connexin function hold significant potential for improving healing outcomes.

### Connexins in skin cancer

The epidermis is a complex tissue composed of various cell types, including keratinocytes, melanocytes, Langerhans cells, and Merkel cells. Among these, melanocytes and keratinocytes are the most well-known for their susceptibility to skin cancers, including melanoma, basal cell carcinoma (BCC), and squamous cell carcinoma (SCC). Numerous factors, including connexins, play roles in the pathogenesis of these cancers (Aasen et al., 2018). The distribution of connexins within the different layers of the epidermis has been described previously. Additionally, their expression varies among different cell types in the skin: Cx26, Cx30, and Cx43 are expressed in keratinocytes, whereas Cx23, Cx26, Cx32, and Cx43 are expressed in melanocytes. (Wong et al., 2016).

Melanocytes, situated in the stratum basale, are responsible for producing melanin pigment to protect the skin from UV radiation (Orellana et al., 2021). Melanoma, the deadliest form of skin cancer, originates from melanocytes and has the potential to metastasize to lymph nodes, lungs, the liver, and the brain (Kircher et al., 2016; Varela-Vázquez et al., 2020). In the context of skin cancer development, the downregulation of Cx26 and Cx43 expression has been linked to processes that promoted metastasis, such as wound healing, epithelial-to-mesenchymal transition (EMT), and cell proliferation (Kiszner et al., 2019). Melanoma cells with low Cx43 levels demonstrated reduced proliferation, tumor size, and metastatic potential, correlating with cytoplasmic localization of Cx43 (Ableser et al., 2014; Tittarelli et al., 2015). Conversely, melanoma cells and endothelial cells surrounding the melanoma cells expressed high levels of Cx26, and increased Cx26 expression correlated with enhanced metastatic potential, possibly facilitating the communication/interaction between melanoma cells and endothelial cells (Saito-Katsuragi et al., 2007). However, other studies have shown high Cx43 expression in metastatic melanoma lesions, suggesting that Cx26 may not be exclusively critical player in this process (Sargen et al., 2013). Furthermore, a study supporting the combined role of Cx26 and Cx43 in melanoma metastasis demonstrated that inhibiting both connexins in zebrafish and chicken embryos impaired cell-cell communication and prevented brain metastatic lesion formation (Stoletov et al., 2013). These findings collectively suggest that differential connexin expression affects intercellular communication and plays a regulatory role in melanoma progression.

Apart from channel activity, connexins can influence multiple signaling pathways that directly regulate cell cycle-related proteins such as cyclins (Cronier et al., 2009). For instance, Cx43 expression suppressed cell proliferation by prolonging the G_2_/M phase through p21 activation in a β-catenin/TCF-dependent manner (Kamei et al., 2003). Additionally, Cx26, Cx30, and Cx43 suppressed the tumor growth through the cAMP signaling pathway, highlighting the complex role of connexins in melanoma biology (Chen et al., 2020).

Differential expression patterns of Cx26, Cx30, and Cx43 have been observed in SCC, a relatively common type of skin cancer with a generally favorable prognosis when compared to melanoma. However, SCC has the potential to metastasize as well (Howell and Ramsey, 2023). Cx26 and Cx30 were upregulated in SCC, whereas Cx43 was either downregulated or remained unaffected (Haass et al., 2006). In contrast, oral SCC exhibited downregulation of Cx26 and upregulation of Cx43, where high Cx43 expression was correlated with poor prognosis, suggesting that connexins may play distinct roles in different subtypes of epithelial cancers (Brockmeyer et al., 2016). Notably, approximately 15% of KID syndrome patients harboring Cx26 mutations developed SCC in skin and oral mucosa, further indicating the role of Cx26 in SCC (Conrado et al., 2007; Taki et al., 2018).

Similar to other skin cancers, Merkel cell carcinoma (MCC), a rare and aggressive cutaneous neuroendocrine tumor, exhibited significantly decreased Cx43 levels across various MCC tumor types. This suggests a potential role for Cx43 dysregulation in MCC pathogenesis (Fernandez-Flores et al., 2020). The differential expression of connexins in diverse cancers indicate their unique functions in the skin. Thus, deciphering the molecular and cellular changes arising from aberrant connexin expression in skin cancers holds promise for the development of novel therapeutic approaches.

## Connexin targeting therapeutics

Connexins have garnered significant research interest due to their involvement in various diseases. Over the years, therapeutic strategies targeting connexin function have emerged, including non-peptide chemicals, peptide mimetics, antibodies, allele-specific siRNAs (AS-siRNAs), and antisense oligodeoxynucleotides (asODNs) (Table 2). Connexin channel inhibitors, in particular, have played a key role in elucidating their function through channel activity modulation. Carbenoxolone (CBX), a non-selective GJ blocker, has been a pillar since 1986 (Davidson et al., 1986). Its ability to reduce fluorescent dye uptake in KID syndrome-associated Cx26 mutant cells (Aypek et al., 2016; Taki et al., 2018) highlighted its potential in investigating hyperactive channel function. However, its non-specificity and side effects limit its therapeutic application (Tovar et al., 2009; Connors, 2012; Buckley et al., 2021). Mefloquine, an antimalarial drug, effectively blocks Cx50 and Cx36 GJ channels (Cruikshank et al., 2004) and showed promise in inhibiting KID syndrome-associated Cx26 mutations (Levit et al., 2015). Its lipophilicity facilitates topical delivery, but its approved indications and potential neuropsychiatric concerns require cautious consideration (Weinke et al., 1991). Boldine, an alkaloid, specifically inhibits Cx43 GJs and holds potential for diabetic wound healing (Hernández-Salinas et al., 2013). Similarly, tonabersat, a small molecule targeting Cx43 hemichannels (Lyon et al., 2020), demonstrated efficacy in reducing inflammation and maintaining vascular integrity in retinal disease models (Mat Nor et al., 2020). These studies showcase the diverse array of connexin inhibitors utilized to investigate connexin channel function. However, further research is warranted to address their specificity and potential side effects in complex biological systems for clinical applicability.

**TABLE 2:** Connexin targeting therapeutics and their applications.

Molecules	Condition	Target	Effect	References
Gap26	Non-healing cutaneous wounds	Cx43 hemichannels	Increased migration rates of keratinocytes and fibroblasts *in vitro*	Wright et al. (2009)
Gap27	Non-healing cutaneous wounds	Cx43 hemichannels	Increased migration rates of keratinocytes and fibroblasts *in vitro*	Wright et al. (2009)
	Corneal wound healing		Accelerated wound closure, *in vitro, ex vivo,* and *in vivo*	Elbadawy et al. (2016)
Peptide5	Ischemia injury	Cx43 hemichannels	Promoted neuronal survival ischemic injury-reperfusion models and spinal cord injuries	Kim et al. (2017)
αCT1	Diabetic foot ulcers, venous leg ulcers	Cx43 ZO-1 binding domain	Accelerated wound closure, re-epithelialization	Ghatnekar et al. (2009), Grek et al. (2015), Ghatnetkar et al. (2015), Montgomery et al. (2021)
			Phase 2/3 clinical trial for surgical wounds (NCT04331080)	
			Terminated phase 3 clinical trial for diabetic foot ulcers (NCT02667327, details unavailable)	
Gap19	Human gingival wounds	Cx43 hemichannels	Faster wound healing	Tarzemany et al. (2017)
abEC1.1	Cx26-G45E KID syndrome mouse model	Cx26 hemichannels	Reduced epidermal thickening and decreased the size and number of keratinocytes in	Peres et al. (2023)
abEC1.1	Cx30-A88V Clouston syndrome mouse model	Cx30 hemichannels	Reduced the proliferation of skin cells and decreased the size of sebaceous glands	Kuang et al. (2020)
AS-siRNAs	KID syndrome cell line model with Cx26-D50N mutation	Cx26 hemichannels	Reduced mutant protein expression and the hyperactive hemichannel activity	Lee et al. (2020)
asODNs	Non-healing corneal wounds		Promoted wound repair and epithelial regeneration	Ormonde et al. (2012)
			Accelerated wound healing in Phase I and II clinical trials (NCT00820196, details unavailable)	

Driven by the non-specific nature of connexin and channel inhibitors, researchers have actively explored novel strategies for selectively targeting specific connexin proteins and their associated channels. The six cysteine residues within the extracellular domains of connexins play a critical role in GJ assembly by forming disulfide bonds with other cysteines. This disulfide exchange is thought to be essential for channel docking and opening. Understanding the function of these cysteines has facilitated the design of peptides that can mimic connexin interactions during GJ channel formation. Additionally, variable amino acids within the extracellular loops influence the specificity of these interactions, enabling the development of connexin-specific peptides (Dahl et al., 1992). Short peptides containing conserved motifs in the extracellular loops involved in junction formation were synthesized (Warner et al., 1995) and peptides containing the SRPTEK motif in the EL2 and amino acids in the putative membrane-spanning region effectively altered GJ formation. Moreover, mimetic peptides containing this motif effectively delayed GJ formation.

Connexin mimetic peptides, Gap26 and Gap27 containing the SHVR and SRPTEK motifs, respectively, were used to investigate the functionality and expression of connexins in vascular cells (Warner et al., 1995). Importantly, these peptides did not impair *de novo* connexin expression or trafficking. Another study examined the potential of these peptides for wound healing by studying their effects on human epidermal keratinocytes and dermal fibroblasts *in vitro*. Blocking GJIC using these peptides increased cell migration rates, suggesting a promising avenue for promotion of wound healing (Wright et al., 2009). Further research revealed that Gap26 and Gap27 achieved their effects by preventing hemichannel docking and promoting cell movement (Lorraine et al., 2015). Specifically, Gap27 was tested as a potential wound healing agent in three settings: primary human corneal epithelial cells (*in vitro*), whole human corneas (*ex vivo*), and a rat wound healing model (*in vivo*). Applying Gap27 accelerated wound closure and corneal cell layering without affecting the release of the inflammatory cytokines IL-6 and TNF-α. After 7 days, Gap27 increased early granulocyte infiltration and the expression of TNF-α and TGF-β, but it did not prevent neovascularization (Elbadawy et al., 2016).

Peptide5, another extensively studied connexin mimetic peptide, targets a region within the EL2 of Cx43 and harbors the SRPTEK motif. At low concentrations, Peptide5 effectively blocked hemichannel opening without disrupting GJIC. However, at higher concentrations, it disrupted the function of pre-existing junctions (O'Carroll et al., 2008). Intriguingly, Cx43 hemichannels have been implicated in secondary lesion spread, a process where the opening of a dysfunctional pore leads to cell death at lesions and exacerbates secondary lesion formation. Peptide5 has been shown to effectively prevent lesion spread and reduce vascular permeability. Furthermore, Peptide5 exhibited anti-inflammatory properties and enhanced neuron survival in various models of brain, retina ischemia-reperfusion injury, and spinal cord injuries (Kim et al., 2017).

αCT1, a Cx43 C-terminal mimetic peptide, effectively inhibits Cx43 hemichannel activity. Harboring the Cx43 zonula occludens-1 (ZO-1) binding domain, αCT1 disrupted their interactions, leading to the sequestration of Cx43 hemichannels from the perinexus regions (Rhett et al., 2011). This consequently reduced the hemichannel density on the plasma membrane. In addition, αCT1 also affected the interaction of Cx43 with other molecules, which in turn altered its phosphorylation status (O'Quinn et al., 2011). αCT1 was shown to hold promise for wound healing. Its application improved the wound closure, promoted the epidermal complexity, decreased the area of granulation tissue, and enhanced the strength and extensibility of the skin in mouse models by attenuation of neutrophil infiltration and changing the epidermal Cx43 organization (Ghatnekar, et al., 2009). Additionally, αCT1 increased the rate of wound closure and reduced inflammation in excisional wounds that were recovered with decreased discoloration (Ongstad et al., 2013). The efficacy and safety of αCT1 was assessed in chronic diabetic wound healing with randomized and multi-center clinical trials. It was observed that the ulcer area was reduced by 94% within 12 weeks of treatment without immunogenic or negative consequences (Grek et al., 2015). In venous leg ulcers, the upregulation of Cx43 expression was observed in the edges of wounds and the incorporation of αCT1 in standard of care treatment greatly reduced the ulcer area in 12 weeks when compared to compression bandage therapy alone (Ghanetkar et al., 2015). The benefit of αCT1 was also evident in human incisional wounds as incisions treated with αCT1 mimetic peptide showed 47% improvement in scar scores at 9 months with progression in scar pigmentation, and roughness of the surface without adverse effects (Grek et al., 2017). The basis for the effectiveness of the αCT1 on the wound was shown to be through the alteration of the organization of collagen bundles in dermal granulation tissue, resulting in its random distribution similar to unwound tissue. This randomness was suggested to interfere with the migration of cells in the wound, accelerating the healing rate (Montgomery et al., 2021). Among Cx43 mimetic peptides only αCT1 (Granexin) is undergoing phase 3 clinical trials for skin wounds without local or systemic adverse effects (Montgomery et al., 2018).

Gap19, derived from the cytoplasmic loop of Cx43, selectively blocks Cx43 hemichannels without hindering GJ closure. This blockade is achieved by preventing interactions between the C-terminus and cytoplasmic loop (Wang et al., 2013). Human gingival wounds that heal faster than skin wounds had distinct hemichannel and GJ plaques where Cx43 hemichannels were implicated in expression of wound healing-associated genes (Tarzemany et al., 2017). Moreover, inhibition of Cx43 hemichannels with Gap19 in human gingival fibroblasts modulated the expression of genes involved in wound healing, leading to faster repair and also suggested targeting connexin hemichannels as an additional component in wound healing process (Tarzemany et al., 2017; Tarzemany et al., 2018).

A monoclonal antibody (mAb), abEC1.1, that targets Cx26 hemichannels was identified in a combinatorial library of human single-chain fragment variable antibodies and characterized using organotypic cultures of mouse cochlea, HeLa, and HaCaT cell lines (Xu et al., 2017). In this study, abEC1.1 effectively inhibited hyperactive Cx26-G45E and Cx26-D50N mutant channel activity. To validate the efficacy of abEC1.1 for the treatment of KID syndrome caused by hyperactive Cx26 mutant channels, Peres et al. employed adeno-associated virus-mediated abEC1.1 monoclonal antibody gene transfer treatment in the mouse model. The treatment effectively blocked Cx26-G45E mutant hemichannel activity in the epidermis, leading to an improvement in skin pathology associated with KID syndrome. Without affecting Cx26 expression in the lesional epidermis, abEC1.1 mAb reduced epidermal thickening and decreased the size and number of keratinocytes. Additionally, abEC1.1 treatment restored the expression of keratins (Peres et al., 2023). Similar to Cx26 KID syndrome model, abEC1.1 also blocked the Cx30-A88V hyperactive hemichannels in Clouston syndrome mouse model (Kuang et al., 2020). The topical or systemic administration of the antibody reduced the proliferation of skin cells and decreased the size of sebaceous glands, suggesting the therapeutic potential of abEC1.1 for treating skin pathologies resulting from hyperactive connexin hemichannels.

AS-RNAi is a highly specific technique that can distinguish between two mRNA sequences differing by just one nucleotide. This strategy holds a promise for silencing mutated alleles that cause dominant inherited diseases. In a landmark study published in 2015, Trochet et al. reported the first clinical trial of AS-RNAi therapy for Pachyonychia congenita, an autosomal dominant condition primarily affecting nails and hand skin (Trochet et al., 2015). The encouraging results have opened up avenues for potential applications in other dominant inherited diseases (Smith et al., 2006; Trochet et al., 2015). Building on this approach, Lee et al. developed AS-siRNAs and applied them to an immortalized KID syndrome cell line (KID-KCs) derived from a patient harboring a heterozygous Cx26-D50N mutation, the most prevalent KID syndrome-associated mutation in the *GJB2* gene, leading to aberrant hemichannel function (Lee et al., 2020). Among several candidates, S7 AS-siRNAs exhibited the most potent knockdown efficacy, inducing a 63% reduction in total Cx26 mRNA levels and a 56% decrease in Cx26 protein expression in KID-KCs without affecting the expression of the wild-type allele. Additionally, neurobiotin uptake assays revealed a significant decline in hemichannel activity following AS-siRNA application (Lee et al., 2020). Further investigation is warranted to fully elucidate the potential of AS-siRNAs on patients, as well as on the expression of other genes to evaluate any potential side effects. Overall, this study offers valuable insights into the development of AS-RNAi as a therapeutic strategy for skin pathologies associated with dominant mutations arising from single nucleotide changes.

Finally, inhibition of Cx43 using asODNs demonstrated efficacy in treatment of wounds. Mori et al. showed that asODN application in mice reduced Cx43 mRNA level, leading to increased proliferation and migration of keratinocytes and fibroblasts, decreased inflammation, and enhanced granulation tissue formation (Mori et al., 2006). Similarly, sustained release of Cx43-asODN via collagen scaffolds improved wound healing in rats (Gilmartin et al., 2016). Moreover, targeting Cx43 expression with Nexagon, a Cx43-asODN developed by Ocunexus, accelerated wound healing in Phase I and II clinical trials (details unavailable). Notably, Ormonde et al. showed Nexagon efficiently promoted wound repair and epithelial regeneration in non-healing corneal wounds, with a single application stimulating early limbal vessel recovery and corneal re-epithelization within 18 h, showing the efficacy of asODN in different types of wounds (Ormonde et al., 2012). Similar to Cx43, Cx26-asODN-coated collagen scaffolds inhibited the rise of Cx26 protein at the edge of excisional wounds and promoted healing in rats, suggesting the potential of asODNs for treating skin conditions with elevated connexin levels (Phillips et al., 2018).

The field of connexin-targeting therapeutics has witnessed significant advancements, including the development of hemichannel/GJ inhibitors, mimetic peptides, allele-specific RNA interference, and asODNs. Recently, specific antibodies such as abEC1.1 have shown promising potential in treating skin pathologies associated with connexin mutations. This progress holds great promise for the development of targeted therapies and novel side-effect-free techniques for diseases resulting from connexin mutations or altered connexin expression.

## Conclusion

The diverse expression patterns of connexins across all epidermal layers and cell types underpin their essential role in maintaining skin health. Moreover, the association of various skin disorders with diverse phenotypes caused by connexin gene mutations supports the importance of each connexin. However, the unique function of each isoform during epidermal homeostasis is not exactly known (Table 1). Functional characterization of individual connexin mutations has provided valuable insights into their effects on protein function and their roles in the skin. These studies reveal that skin-associated connexin mutations can disrupt protein function in several ways, depending on the type and location of the alteration within the polypeptide sequence. These disruptions can include: defects in the biosynthetic pathway, impaired GJIC, dominant-negative effects on wild-type connexins, and the formation of hyperactive hemichannels (Figure 4). Impaired GJIC, either due to the mutant connexins themselves or their dominant-negative effect on wild-type connexins, can alter the type and extent of communication between cells, potentially affecting proliferation and differentiation processes. Furthermore, the generation of hyperactive hemichannels on the plasma membrane can facilitate uncontrolled molecular exchange between the cytoplasm and the extracellular environment, particularly for signaling molecules such as Ca^2+^ and ATP. Increased intracellular Ca^2+^ levels can modify the activity of several Ca^2+^-dependent signaling processes, interfering with epidermal homeostasis. Hemichannels can also release molecules like ATP into the extracellular environment. This leakage can not only promote cell death but also influence the behavior of surrounding cells by acting as paracrine signals that activate purinergic receptors or other mechanisms, ultimately disrupting cell function. Finally, the retention of mutant proteins in the cytosol can impair cellular structures and alter the expression of molecules such as AP-1 or TGF-β1, which are involved in maintaining keratinocyte homeostasis. Additionally, connexin expression is dysregulated in various skin conditions such as psoriasis, chronic wounds, and cancers. Ultimately, these disruptions interfere with keratinocyte proliferation and differentiation, thereby contributing to the development of various epidermal disorders. While the general impact of connexin mutations or dysregulated expression on epidermal homeostasis has been started to be understood, the exact underlying mechanisms and the effects of connexin alteration on cellular processes that lead to epidermal changes remain largely unknown. Thus, deciphering these molecular and cellular changes arising from abnormal connexin function involved in keratinocyte proliferation and differentiation processes is crucial for better understanding the pathogenesis of each skin condition, potentially leading to the development of more precise therapies for treatment or symptom management.
